# Assessment of Masseter Muscle Appearance and Thickness in Edentulous and Dentate Patients by Ultrasonography

**DOI:** 10.2174/1745017901814010723

**Published:** 2018-09-28

**Authors:** Meltem Mayil, Gaye Keser, Arzu Demir, Filiz Namdar Pekiner

**Affiliations:** Department of Oral and Maxillofacial Radiology, Marmara University, Faculty of Dentistry, Istanbul, Turkey

**Keywords:** Ultrasonographic appearances, Masseter muscle, Dentate group, Edentulous patients, Echogenic type, Temporomandibular disorder

## Abstract

**Objectives:**

The purpose of the present study was to examine ultrasonographic appearances of Masseter Muscle (MM) in dentate and edentulous patients without Temporomandibular Disorder (TMD).

**Materials and Methods:**

The thickness of the MM in 25 dentate (mean age: 30,68 ± 10,49) and 24 edentulous (mean age: 61,46 ± 9,71) patients, who visited routine dental examination, was measured at rest and at maximum contraction bilaterally. Examinations were performed using an Aloka Prosound α6 (Hitachi Aloka Medical Systems, Tokyo, Japan) equipped with an 8 MHz-wide bandwidth linear active matrix transducer (ranging from 1 to 15 MHz). The visibility and width of the internal echogenic bands of the MM were also assessed and the muscle appearance was classified as I of III types. Type I, characterized by the clear visibility of the fine bands; Type II, thickening echogenicity of the bands; Type III, disappearance or reduction in a number of the bands.

**Results:**

MM thickness at rest and contraction in the dentate group were significantly higher than the edentulous group (*p* <0.05). Type I was the most common echogenic type in both dentate (right:16 (64%), left; 15 (60%)) and edentulous patients (right; 22 (91.7%), left; 18 (75%)). In a dentate group, type II was significantly higher than the edentulous group in both the right and left sides (*p* <0.05; *p* <0.01, respectively). Age and gender seemed to have no significant effect on the echogenic type (*p* ˃0.05).

**Conclusion:**

There were significant differences in the thickness at rest and contraction between the dentate and edentulous groups. It was clarified that ultrasonographic features of the MM in dentate and edentulous patients were different.

## 1 INTRODUCTION

Relationships between edentulism, temporomandibular joint, musculature and the nervous system are important. The symmetry of muscle size on the right and left side and morphology within each individual are significant when the literature is reviewed. A broad range of factors, such as skeletal size, age, masticatory habits and general health are related to variation between individuals [1-4].

Changes in the muscle fiber size and composition, which in turn will increase the strength of the muscle and the resistance to fatigue may be a result of intensive use of any skeletal muscle. This is also relevant for the masticatory muscles. Therefore, it could be expected that this prolonged bilateral difference in the activity level of the masticatory muscles may work as an asymmetric training stimulation which results in differences in the thickness of the muscles. However, the masticatory muscles are also involved in functions that are not necessarily related only to mastication, and it is unknown that if previously observed bilateral differences in the activity level of the masticatory muscles are enough to create detectable changes in the muscle thickness [5-8].

A negative impact on the macro- and microscopic structure of the chewing muscles is observed with the patient’s age and years of the edentulism [9]. Radiographic density and the cross-sectional area of the masseter and medial pterygoid muscle decrease with age and these effects are significantly provoked by edentulism [10]. The masseter muscle is one of the dominant muscles that provide the force necessary to chew efficiently. Mastication can occur bilaterally, yet 78% of observed subjects have a preferred side where the majority of chewing occurs. This is generally the side with the greatest number of tooth contacts during lateral glide [11, 12].

Unilateral partial edentulous subjects mostly use the dentate side, and this is where the majority of the chewing occurs [13]. The weight of the masseter muscle was significantly reduced in a study by Urushiyama *et al*. [14]^,^ where the change was made from a hard to a soft texture diet in an animal model. Mice fed with a soft diet lost 19% of the muscle weight within 1 week. For complete denture wearers who often compensate for the lack of masticatory ability by changing to a softer diet, these findings are particularly relevant [15]. The observed atrophy of the muscle tissues in edentulous patients with conventional complete dentures may, therefore, be caused by a “de-training” effect.

Masseter (MM) is a thick, quadrilateral muscle, consisting of superficial and deep portions. The superficial portion, the larger area, arises with tendinous aponeurosis from the zygomatic process of maxilla and its fibers pass downward and backward attaches lateral surface of the ramus of mandible. The deep portion is much smaller and arises from the posterior lower border of the zygomatic arch; and its fibers pass downward and forward, to attach onto the upper half of the ramus and coronoid process [3, 16, 17].

With various imaging techniques including ultrasound scanning, Computerized Tomography (CT) and Magnetic Resonance İmaging (MRI), masseter muscle thickness has been measured. Ultrasound imaging (US) is particularly suitable for imaging superficial structures of the head and neck region. It is appropriate for larger scale studies, due to its numerous advantages comparing to CT and MRI [18]. Without the use of ionizing radiation, US allows an economical method to measure muscle thickness [18-22]. Also, the ultrasound equipment is accurate for soft tissues assessment [21,23]. Therefore, US is a simple technique with a rapid diagnosis, non-invasive and does not use ionizing radiation. This technique allows the clinician to measure accurately the thickness of the masseter muscle [20, 21,24]. As a choice of US in determining the MM thickness was guided by the recommendations of previous studies, which described reliability, accuracy and advantages of the ultrasound methods comparing with MRI and CT [19, 20, 22,25].

The purpose of this study was to assess the masseter muscle appearance and thicknesses while the teeth were occluding relaxed and contracted during maximal clenching in dentate healthy subjects and in edentulous patients by ultrasonography. In addition, the effect of age and gender on muscle appearance and thickness in the two group were evaluated.

## 2 MATERIALS AND METHODS

Twenty-five dentate (13 female, 12 male) and twenty- four (12 female, 12 male) edentulous patients without TMD were enrolled in this study. The age range was from 22 to 55 (mean: 30,68±10,49) for the dentate group and from 41 to 81 (mean: 61,46±9,71) years old for the edentulous group. All subjects were continuously selected from patients who visited Marmara University, Faculty of Dentistry, Department of Oral Diagnosis and Radiology for several complaints. The edentulous patients were selected as missing all teeth and wearing a complete dental prosthesis at least five years, the dentate patients were selected as missing no teeth in their mouth. The patients were selected on the basis of the following criteria: (1) no bony changes that effect masticatory muscle function on conventional radiographs of maxilla, mandible and TMJ region (2) no history of masticatory muscle pain at least 3 months or more before they visited our clinic; (3) no pain with palpation on masticatory muscle during clinical examination (4) no deviation or limitation while mouth opening (5) no disease which affects muscle structure (submucous fibrosis, dermatomyositis, myasthenia gravis... *etc*). The study protocol numbered as 09.2015.129 was approved by Non-invasive Clinical Research Ethics Committee, Marmara University Faculty of Medicine. The patients were informed about the study procedure and informed consent was received.

**Fig. (1):**
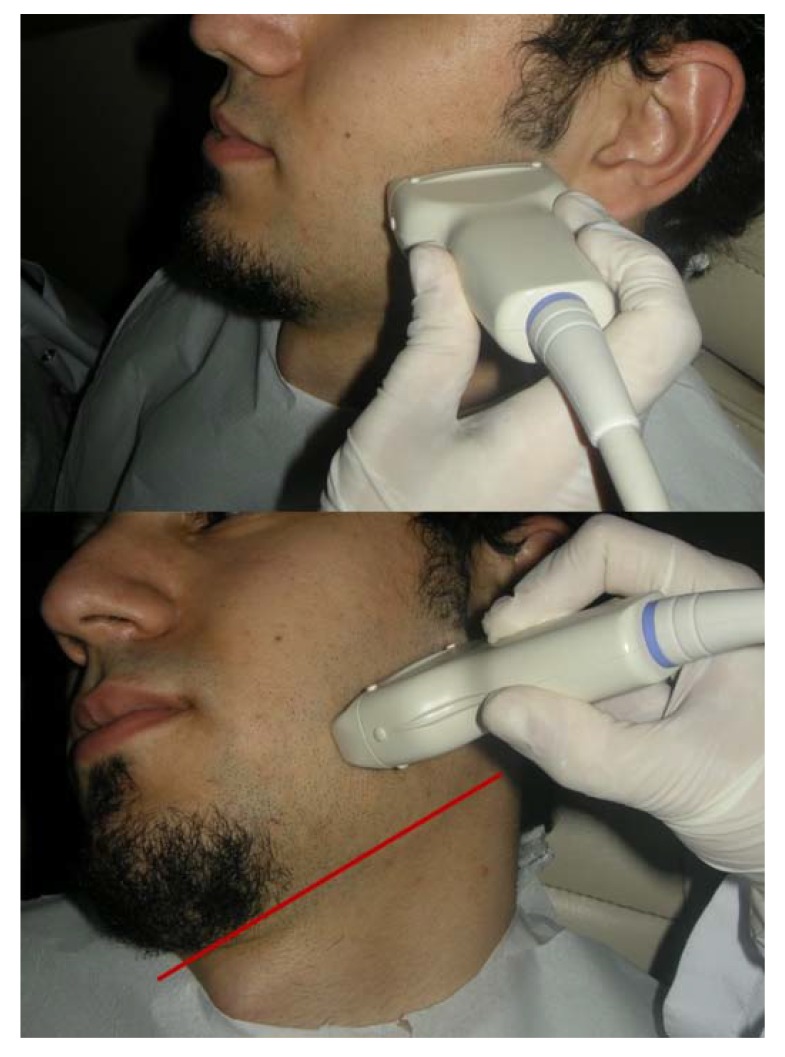
US examination of MM with the transducer positioned perpendicular to muscle surface and aproximately 2.5 cm above the inferior border of mandible (The red line shows the inferior border of mandible).

Each patient was seated in an upright position with the head in the natural position. At first, the patients were asked to be relaxed and MM thicknesses were measured bilaterally. At the second stage, the patient was asked to clench their teeth and MM thicknesses were measured again bilaterally. The measurements for each group were obtained from the thickest part of MM. The thickness was defined as the maximal distance between the outer fascia of the muscle and the lateral surface of the ramus. While doing measurements, both sides of the masseter muscle were scanned perpendicular to the anterior border of the muscle and the surface of the mandibular ramus at approximately 2.5 cm above the inferior border of the mandible with minimum pressure to obtain the muscle image as thick as possible (Fig. **1**). Examinations were performed by using an Aloka Prosound α6 (Hitachi Aloka Medical Systems, Tokyo, Japan) equipped with an 8 MHz-wide bandwidth linear active matrix transducer (ranging from 1 to 15 MHz). The ultrasonograms were obtained with MM mode and an image depth of 3.5 cm, echo gain was 80-90 dB. After thickness measurement, the appearance of MM was also assessed by considering visibility and width of the internal echogenic bands and the muscle appearance were classified into three types.

**Fig. (2):**
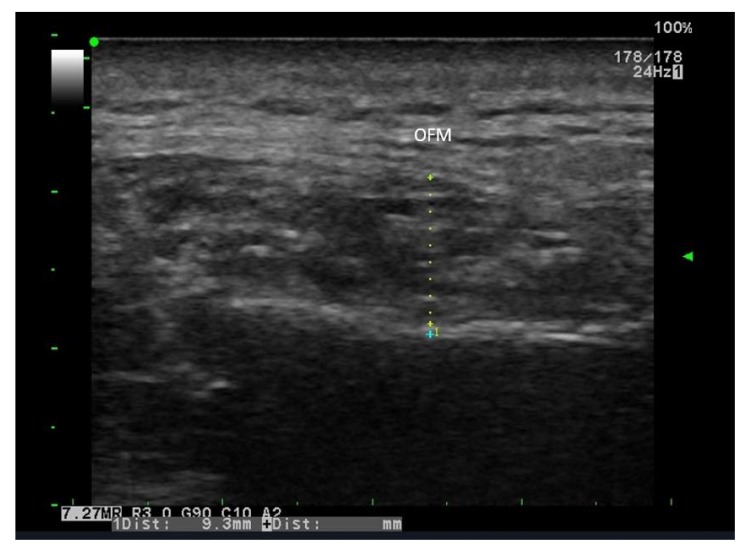
Measurement of MM thickness from the Outer Fascia of the Masseter Muscle (OFM) to the Lateral Surface of the Ramus (LSM) and evaluation of internal sonotype I; characterized by the clear visibility of the fine bands.

**Type I**: Characterized by the clear visibility of the transverse fine bands at the middle depth of the muscle (Fig. **2**)

**Fig. (3):**
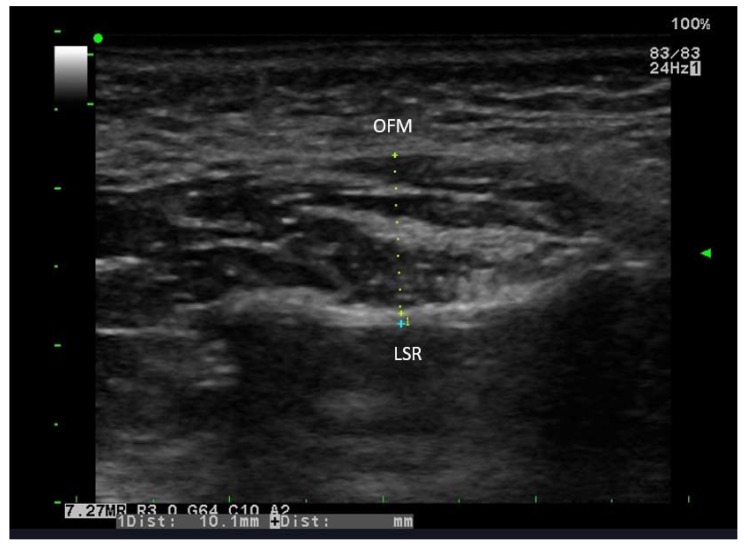
Measurement of MM thickness from the Outer Fascia of the Muscle (OFM) to the Lateral Surface of the Ramus (LSR) and evaluation of internal sonotype II; characterized by thickening echo-intensity echogenicity of the bands.

**Type II**: Thickening and the weakened echogenicity of the transverse fine bands and several short bands (Fig. **3**)

**Fig. (4):**
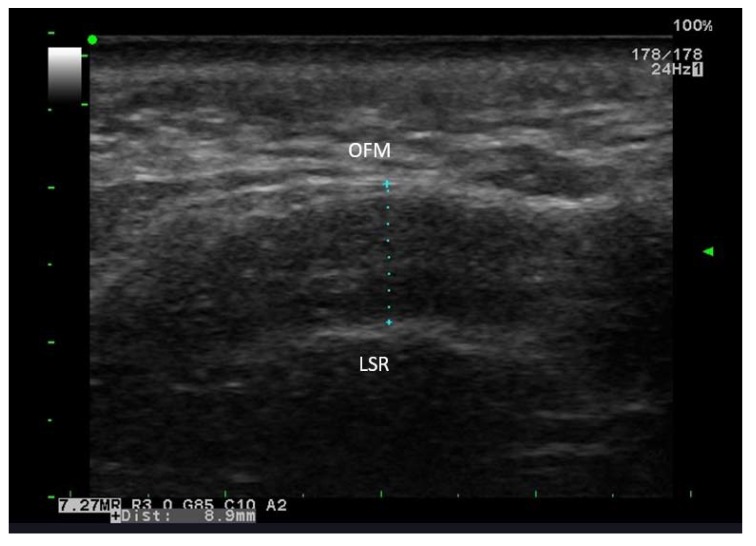
Measurement of MM thickness from the outer fascia of the masseter muscle (OFM) to the lateral surface of the ramus (LSR) and evaluation of internal sonotype III; characterized by disappearance or reduction in number of the bands.

**Type III**: Disappearance or reduction in a number of the several short bands without a transverse band ( Fig. **4**).

All of the evaluation and measurements were performed by three clinicians together (M.M., G.K., and A.D.), who had sufficient experience in the interpretation of ultrasonography. The clinicians had been trained and calibrated by a specialist who has been working in Oral Diagnosis and Radiology for fifteen years (F.N.P). Calibration trials were performed initially to ensure an inter-examiner consistency of at least 85% in the recording. For calibration, 12 patients were evaluated and not included in the main study. The inter-examiner agreement was measured by Cohen’s Kappa statistic.

### 2.1 Statistical Analysis

All analyses were performed in IBM *SPSS* Statistics 22 (IBM *SPSS*, Turkey) environment. The student t-test was used for comparison of descriptive statistics (mean, standard deviation) and quantitative data with normal distribution between two groups. Mann Whitney U test was used for parameters showing abnormal distribution between 2 groups. Kruskal Wallis test was used for the comparison between the groups of parameters that were not normally distributed. The Chi-squared test, Fisher’s exact chi-squared test and Continuity (Yates) correction were used for comparing qualitative data. *p* <0.05 was accepted for as significance level.

## 3 RESULTS

The mean age of patients was 30,68 ± 10,49 for the dentate group and 61,46 ± 9,71 for the edentulous patients. The mean age of the dentate group was significantly lower than the edentulous group (*p* <0.01).

The means of MM thicknesses for each group at rest and contraction stages are seen in Table **1**. The means of right MM thicknesses of dentate group at rest and at contraction were higher than the edentulous group (*p* <0.01). The means of left MM thicknesses of dentate group at rest and contraction were also higher than edentulous group (*p* <0.05; *p* <0.01).

The means of increases of MM thicknesses while passing from rest to contraction stage are seen in Table **1** for each group. This increase was statistically significant within the groups (*p* <0.01) but a comparison of increases among the groups were not (*p* >0.05).

In dentate and edentulous groups, no significant relationship was found between age and both relaxed MM thickness and contracted MM thickness (*p* >0.05) (Table **2**).

In a dentate group, the means of right MM thicknesses at rest and contraction were higher in male than female (*p* <0.05). On the left side, the mean of MM thicknesses at rest was higher in male than female (*p*:0.049; *p* <0.05) but there was no significant difference at contraction stage (*p* >0.05). Among males and females, there was no significant difference between the means of increases of MM thicknesses while passing from rest to contraction stage (*p* >0.05) both on left and right sides (Table **3**).

In an edentulous group, both on left and right sides among the genders, there was no significant difference between means of right MM thicknesses at rest and contraction (*p*>0.05). Among males and females, there was no significant difference between the means of increases of MM thicknesses while passing from rest to contraction stage (*p*>0.05) on both left and right sides (Table **3**).

Type I was the most common band echogenicity type in the dentate group for both left (%60) and right (% 64) sides, also in edentulous group for both left (75%) and right (91.7%) sides. There was a statistically significant difference on the right side according to band echogenicity type between dentate and edentulous groups (*p* <0.05). On the right side in dentate group (24%), band echogenicity Type II rate was significantly higher than edentulous group (0%) (*p* <0.05). Type I rate in dentate group (64%) was significantly lower than edentulous group (91.7%) on the right side (Table **4**).

There was also a statistically significant difference on the left side according to the muscle appearance between dentate and edentulous groups (*p* <0.01). The origin of the significance was same as on the right side, that band echogenicity Type II rate in the dentate group (32%) was significantly higher than edentulous group (0%) on left side (*p* <0.01). A statistically significant difference was not found between I and III band echogenicity types in dentate and edentulous groups on the left side (*p* >0.05) (Table **5**).

No effect of age and gender on the muscle appearance was found both in each dentate and edentulous when evaluated within each group (*p*>0.05) (Tables **5** and **6**).

## 4 DISCUSSION

In the current study, MM was assessed with ultrasonography as described by Ariji *et al*., [26]. Systemic diseases, loss of dental structure, pathology and facial musculature pain, age and gender are the factors associated with masticatory performance [12,27-29]. Therefore, patients with systemic diseases and muscle pathologies were excluded from our study.

Ultrasonography had been used to measure thickness and evaluate the internal appearance of MM in previous studies and its reliability has already been confirmed [24,30]. Egwu *et al.* [23] used US for evaluating MM and showed that thickness at rest stage was significantly lower than the contraction stage as same as in our study. However, the cause or mechanism of thickening has not been well described and there could be two possibilities for the increase in thickness. When a muscle is contracted, the muscle fiber filament slides on each other and an increase in fiber diameter cause thickening. Ariji *et al*. [26] assumed that this changes based on sliding could be observed concomitant with the beginning of contraction. Another reason could be an edematous change of muscle. The thickening of the MM by the clenching of teeth was also confirmed by an experimental study [24].

It was assumed that chewing with removable prostheses might limit the MM thickness. The reasons for this limitation were explained as the perception of displacement or loosening of the denture during unfavourable loading and the discomfort of food getting caught underneath the denture base. Besides, a previous fracture experience of the prosthesis might limit the functional benefit [23,31].

Kubota *et al*. [32] found that MM thickness as 15.8.0 ± 3.0 mm at rest, and 16.7.0 ± 2.7 mm at contraction in healthy male patients. MM thickness at rest was reported 13.41 ± 3.09 mm and at contraction 17.03 ± 3.50 mm by Egwu *et al*. [23] in healthy patients. In their study, Bakke *et al*. [24] observed that in healthy adults, thickness of the masseter muscle in contraction was strongly correlated with the number of teeth in contact. The study was enrolled in a female subject group and the MM thickness has been found 12.6 ± 1.8.

These values are in accordance with the study of Satiroglu *et al*. [22] who reported MM thickness as 13.56 ± 1.95 mm and 14.57 ± 1.83 mm at rest and contraction, respectively in a Turkish subpopulation. Our dentate group findings supported these values through with the slight environmental deviation (11.08 ± 1.92 at rest and 13.08 ± 2.08 at contracted), and with an increased ratio of 13.53%. On the contrary, Liao and Lo. [33] reported relatively lower MM thickness measurements of the patients without TMD as 9.0 ± 1.9 mm at rest and 11.8 ± 2.8 mm at contraction with an increased ratio of 33% and they also stated that genders should be considered in clinical judgement for evaluating MM thickness. Ariji *et al.* [26] stated that MM thickness at rest as 8.28 ± 1.73 mm for their healthy group. The increased ratio of 38% of their study was similar to Liao and Lo‘s results [33]. Kamala *et al*. [34] supported these low values with 7.75 ± 0.79 mm at rest and 10.15 ± 0.69 mm at contraction in their healthy control group. Similarly, our edentulous group findings were significantly lower than the dentate group (9.40 ± 1.90 at rest and 10.77 ± 1.77 at contracted) with an increased ratio of 14.36%. There was no statistical significance between the increased ratios of edentulous and dentate groups in this study but our ratio values were lower than Ariji *et al.*, [26] and Liao and Lo [33].

None of the previous studies mentioned that whether their patient's groups had been dentate or edentulous. MM thickness values might be effected from the differences of dentition and configuration of the investigated groups eventually. These discrepancies in thickness may also be based on the differences in the measurement methods. For example in the study of Bennington *et al*., [25], the thickness was measured at the anteroposterior midpoint of the muscle belly. In our study and the previous studies; measurements were performed at the point of estimated maximum thickness of muscle [19, 20]. Bakke *et al.* [30] also measured the thickness at the anteroposterior midpoint but they estimated the bulkiest level of the muscle. On the other hand, these variations in measurements of different populations may be related with racial perspectives and the relative indulgence in masticatory activities may lead to the tendency of adaptive increase in muscle. It may also be associated with the orientation and size of the muscle fibres that could have genetic and environmental backdrop [23].

Bennington *et al*. [25] reported that male muscle measurements at contraction tended to be larger than female; (MM thickness at contracted 11.1 ± 1.3 mm and 9.5 ± 1.2 mm for males and females respectively). Liao and Lo [33], Egwu *et al*., [23] and Kiliaridis and Kalebo [20] stated that MM thickness at rest and maximal occlusion was higher in male than female patients. Similarly in our study; dentate group values of MM thicknesses at rest and contraction were also higher in male than female except contracted MM thickness values of left side. But in edentulous group, there was no statistical significance among the genders between MM thicknesses both at rest or contraction stages.

In a comparison study of Müller *et al.* [31], the lowest chewing performance was found in the conventional complete denture prosthesis group. However, the dentate control-group showed a significantly better masticatory efficiency than all other groups which were implant-supported fixed or removable dental prosthesis. MM thickness was found the thinnest in the conventional complete dental prosthesis group and the thickest in the dentate control-group in the same study. In our study dentate and conventional complete prosthesis, groups were also compared and MM thickness on both right and left side was found significantly larger in a dentate group than edentulous group, supportively. Schimmel *et al*., [35] also presented a case of a patient who had regained MM thickness after the stabilization of the lower denture with two implants.

An expressive correlation has been shown to exist between age, gender and thickness of the masseter muscle. It has been reported to be greater in men and elderly individuals [21,36]. In a study conducted by Radsheer *et al*., [19], it was reported that thickness of the masseter muscle decreases with age in both sexes. The similar results were portrayed in studies conducted by Kiliaridis & Kalebo [20] and Hernandez *et al*. [37] in relaxed and/or clenched positions of the masseter muscle.

Giving quantitative information about the functional capacity of the muscle, ultrasonography was used to measure the thickness of the masseter muscle [38]. It was reported that the cross-sectional area of the masticatory muscles had reduced in edentulous patients compared with the dentate group regardless of age [10]. Müller *et al*., [31] confirmed that atrophy of the related muscles could have seen as a result of a prolonged low activity. The reduced radiographic density of the masticatory muscles was also shown with computed tomography, after a long time of wearing conventional complete dentures [20].

The internal ultrasonographic features of the MM were also evaluated in our study and the echogenic echogenicity of bands were detected with less echogenicity (Type III) in an edentulous group than the dentate group. In addition, in our study, there was no statistically significant relationship neither between age and MM thickness nor between age and the band echogenicity of MM. In consideration of the echogenic bands correspond to the internal fascia, tendon or collagen fibers of the muscle; it might be mentioned that in the atrophic muscle these bands tend to quietly diminish or disappear. It is known that the echogenicity on US images are weaker when the differences in impedance between tissues are lesser. As sound waves are reflected from the boundaries between tissues with different acoustic impedance. Thus, in the studies conducted by Ariji *et al*., [26,39], less echogenicity is suggested to be a consequence of the reduction of the difference in acoustic impedance between tissues which contact each other.

Although the current study demonstrated that MM thickness and muscle echogenicity were significantly different between dentate and edentulous patients, with the conceptual limitations as small sample size. Furthermore; ultrasonography can be used to evaluate the features of MM safely. The internal ultrasonographic features of the muscles would lead the clinicians to detect the pathologic state of the muscles. Further studies with larger sample size are needed to detect pathologic changes of the inner structure of masticatory muscles on US views.

## CONCLUSION

In our study, MM thickness at rest and contraction in the dentate group were significantly higher than the edentulous group (*p*<0.05). Age and gender seemed to have no significant effect on the echogenic type (*p*˃0.05). It was clarified that ultrasonographic features of the MM in dentate and edentulous patients were different. In summary, the results of this study indicate that ultrasonography is a reproducible method for measuring masseter muscle thickness yet there is a need for standardization of methods and parameters to be recorded and future studies with a larger sample are needed for more accurate results.

## ETHICS APPROVAL AND CONSENT TO PARTICIPATE

The study protocol numbered as 09.2015.129 was approved by Non-invasive Clinical Research Ethics Committee, Marmara University Faculty of Medicine.

## HUMAN AND ANIMAL RIGHTS

No animals were used in this research. All research procedures followed were in accordance with the ethical standards of the committee responsible for human experimentation (institutional and national), and with the Helsinki Declaration of 1975, as revised in 2008 (http://www.wma.net/en/20activities/10ethics/10helsinki/).

## Figures and Tables

**Table 1:** Comparision of MM thickess of dentate and edentulous group.

–	**Dentate**	**Edentulous**	***p***
	**Mean±SD**	**Mean±SD**	
**Right**	–	–	–
**Relaxed**	11,58±2,18	9,43±1,98	^1^0,001**
**Contracted**	13,36±2,31	10,81±1,49	^1^0,001**
**Difference**	1,78±1,2 (1,8)	1,38±1,45 (1,65)	**^2^0,617**
**^3^*p***	0,001**	0,001**	–
**Left**	–	–	–
**Relaxed**	10,58±1,66	9,37±1,82	^1^0,019*
**Contracted**	12,81±1,85	10,74±2,06	^1^0,001**
**Difference**	1,23±2,06 (1,2)	1,32±2,38 (1,45)	**^2^0,818**
**^3^*p***	0,001**	0,001**	–

^1^Student t Test ^2^Mann Whitney U Test ^3^Paired t Test **p<0.01 *p<0.05

**Table 2:** Corelation of MM thickness with age in dentate and edentulous groups.

–	**Age**	
	**r**	***p***
**Dentate**	–	–
**Right Relaxed**	0,075	0,721
**Left Relaxed**	-0,047	0,822
**Right Contracted**	-0,166	0,428
**Left Contracted**	-0,171	0,414
**Edentulous**	–	–
**Right Relaxed**	0,219	0,304
**Left Relaxed**	0,241	0,256
**Right Contracted**	0,150	0,625
**Left Contracted**	0,178	0,406

Pearson Correlation

**Table 3:** Evaluation of effect of gender on MM thickness in gropus.

–		**Female**	**Male**	***p***
		**Mean ± SD (Median)**	**Mean ± SD (Median)**	
**Dentate**	**Right**	–	–	–
	**Relaxed**	10,67±1,94	12,57±2,05	^1^0,026*
	**Contracted**	12,42±1,83	14,39±2,4	^1^0,029*
	**Difference**	1,75±1,48 (1,7)	1,83±0,86 (1,85)	**^2^0,428**
	**^3^*p***	0,001**	0,001**	–
	**Left**	–	–	–
	**Relaxed**	9,97±1,71	11,24±1,38	^1^0,049*
	**Contracted**	12,52±1,73	13,13±1,99	**^1^0,427**
	**Difference**	1,85±1,82 (1,7)	0,56±2,18 (0,7)	**^2^0,173**
	**^3^*p***	0,001**	0,003**	–
**Edentulous**	**Right**	–	–	–
	**Relaxed**	9,2±2,18	9,65±1,82	**^1^0,589**
	**Contracted**	10,28±1,31	11,34±1,53	**^1^0,079**
	**Difference**	1,08±1,5 (1,15)	1,69±1,38 (1,9)	**^2^0,285**
	**^3^*p***	0,031*	0,001**	–
	**Left**	–	–	–
	**Relaxed**	8,93±1,85	9,8±1,76	**^1^0,252**
	**Contracted**	10,23±1,5	11,26±2,46	**^1^0,227**
	**Differrence**	1,03±2,31 (1,45)	1,61±2,52 (1,75)	**^2^0,729**
	**^3^*p***	0,006**	0,004**	–

^1^Student t Test ^2^Mann Whitney U Test ^3^Paired t Test **p<0.01 *p<0.05

**Table 4:** Evaluation of dentate and edentulous groups according to the muscle appearance.

–	**Dentate**	**Edentulous**	***p***
	**n (%)**	**n (%)**	
**Right**	–	–	–
**Type I** **Type II**	16 (64%) 6 (24%)	22 (91,7%) 0 (0%)	0,028*
**Type III**	3 (12%)	2 (8,3%)	
**Left**	–	–	–
**Type I** **Type II**	15 (60%) 8 (32%)	18 (75%) 0 (0%)	0,006**
**Type III**	2 (8%)	6 (25%)	

Chi-square Test **p<0.01 *p<0.05

**Table 5:** Evaluation of effect of age on the muscle appearance in groups.

–		**Age**					***p***
		**III**		**II**		**I**	
		**Mean±SD**		**Mean±SD**		**Mean±SD**	
**Right**	**Dentate**	35±17,44 (27)	34±12,2 (30)		28,63±8,55 (24,5)		**^1^0,572**
	**Edentulous**	52±15,56 (52)	-		62,32±9,08 (62,5)		**^2^0,250**
**Left**	**Dentate**	41,5±19,1 (41,5)	33,13±10,6 (30)		27,93±8,84 (24)		**^1^0,110**
	**Edentulous**	62,67±13,23 (67)	-		61,06±8,68 (60,5)		**^2^0,463**

^1^Kruskal Wallis Test ^2^Mann-Whitney U Test

**Table 6:** Evaluation of effect of gender on the muscle appearance in groups.

–		**Female**	**Male**	**p**
		**n (%)**	**n (%)**	
**Dentate**	**Right**	–	–	–
	**III**	0 (0%)	3 (25%)	**^1^0,143**
	**II**	4 (30,8%)	2 (16,7%)	
	**I**	9 (69,2%)	7 (58,3%)	
	**Left**	–	–	–
	**III**	0 (0%)	2 (16,7%)	**^1^0,133**
	**II**	6 (46,2%)	2 (16,7%)	
	**I**	7 (53,8%)	8 (66,7%)	
**Edentulous**	**Right**	–	–	–
	**III**	0 (0%)	2 (16,7%)	**^2^0,478**
	**I**	12 (100%)	10 (83,3%)	
	**Left**	–	–	–
	**III**	3 (25%)	3 (25%)	**^2^1,000**
	**I**	9 (75%)	9 (75%)	

^1^Chi-square Test ^2^Fisher’s Exact Test
